# Prevalence of workplace bullying among medical students: A meta-analysis and systematic review protocol

**DOI:** 10.1371/journal.pone.0310076

**Published:** 2025-02-13

**Authors:** Hancong Li, Mingchun Mu, Yan He, Jinjin Wang, Zhaolun Cai, Haitao Tang, Bo Zhang, Han Luo, Wen Zeng

**Affiliations:** 1 Department of Thyroid and Parathyroid Surgery, West China Hospital, Sichuan University, Chengdu, Sichuan, China; 2 Gastric Cancer Center, West China Hospital, Sichuan University, Chengdu, Sichuan, China; 3 Department of Biotherapy, Cancer Center, West China Hospital, Sichuan University, Chengdu, Sichuan, China; 4 Department of Hematology, West China Hospital, Sichuan University, Chengdu, Sichuan, China; 5 Department of Postgraduate Students, West China School of Medicine, Sichuan University, Chengdu, Sichuan, China; Universiti Pertahanan Nasional Malaysia, MALAYSIA

## Abstract

Workplace bullying is a significant issue impacting various professions, including the healthcare sector. This study presents a meta-analysis aimed at assessing the global prevalence of workplace bullying among medical students and identifying potential influencing factors. Previous research suggests that medical students experience higher rates of bullying compared to senior doctors. However, no worldwide meta-analysis has been conducted on this topic. By utilizing a reliable measurement tool, this study will offer a comprehensive analysis of the prevalence of workplace bullying among medical students. The findings are intended to inform the development of strategies to address this issue and enhance the well-being and professional development of medical students worldwide.

## Introduction

Workplace bullying is a widespread issue encompassing various forms of mistreatment and hostile behavior, including abusive supervision, incivility, bullying/mobbing, harassment, victimization, emotional abuse, ostracism, and social undermining [1]. Although definitions of workplace bullying vary across studies [2], it is widely recognized as a significant global public health concern that negatively impacts physical and mental well-being as well as occupational functionality [3]. Reports indicate that a substantial proportion of workers across different occupations experience workplace bullying, with prevalence rates ranging from 10% to 20% [4–6].

However, the healthcare setting appears to be particularly affected by workplace bullying. For instance, Chambers *et al*. reported a 38% incidence of workplace bullying among senior doctors in New Zealand [7], while a meta-analysis of 45 studies found an aggregate prevalence of 26.3% among healthcare employees [8]. Additionally, a summary review by Goh *et al*. indicated that at least one-quarter of the nurse population is affected by workplace bullying [9]. Despite variations in reported data, workplace bullying is a pervasive issue in the healthcare industry, which may be attributed to factors such as the emotional nature of healthcare work, conflicting priorities of multidisciplinary teams, and rigid hierarchical structures among healthcare worker [8, 10].

Medical students, occupying the lowest position within the medical hierarchy and being in the preliminary stage of their medical careers, are reported to experience particularly severe forms of bullying during clinical practice [11–13]. A multicenter study involving over 2300 students from 16 American medical schools found 85% experienced harassment or belittling, with 40% experiencing both [14]. Similarly, a recent French epidemiological study reported that 41.7% of medical students and young doctors experienced workplace bullying [15]. In China, a cross-sectional survey of 1,877 resident doctors from 12 hospitals across seven administrative regions found a 51.4% rate of workplace bullying [16]. The severity of workplace bullying among medical students or junior doctors is notably higher than among senior doctors, with some studies reporting prevalence rates exceeding 60% [17–19]. Workplace bullying adversely affects the psychological health and professional development of medical students, leading to decreased confidence in clinical skills, career dissatisfaction, depressive symptoms, burnout, and suicidal ideation [20]. Addressing these issues is crucial for maintaining the morale of the medical workforce and improving recruitment and retention in the profession [11].

Despite the prevalence of workplace bullying among medical students, no global meta-analysis has been conducted on this topic. A previous meta-analysis has reported the prevalence of harassment and discrimination in medical training to be 59.4%, but this study was conducted over a decade ago and showed variations in definitions and participant groups [21]. A more recent meta-analysis by Gianakos *et al*. in 2022 focused solely on surgical residency trainees [22]. Additionally, these studies did not adopt uniform measurement scales, relying instead on pooled prevalence data, which may lead to significant heterogeneity and compromise the robustness of the findings.

Given the varied concepts of workplace bullying and diverse investigation methods, uncertainty remains regarding the overall prevalence of workplace bullying among medical students. Although some small-scale research has been performed in certain hospitals around the world, a comprehensive meta-analysis and systematic review using a reliable and widely recognized tool are necessary to accurately assess the overall prevalence of workplace bullying among medical students.

### Objectives

The proposed systematic review aims to address the following questions:

1. To estimate the global prevalence of workplace bullying among medical students based on uniform diagnostic criteria.
2. To identify and analyze the potential influencing factors affecting workplace bullying among medical students worldwide.

## Method

### Registration

This protocol has been registered on the International prospective register of systematic reviews (PROSPERO) platform (registration number: CRD42023407078 https://www.crd.york.ac.uk/PROSPERO/). Any deviations from this protocol will be noted in the final manuscript. Accordingly, we will update the PROSPERO record. The protocol will be conducted according to the Preferred Reporting Items for Systematic reviews and Meta-Analyses Protocols (PRISMA-P) checklist (**S1 File)** and the MOOSE statement [23, 24].

### Ethics and dissemination

Ethics approval is not required for this protocol and systematic review, as they will involve only summarized and anonymous data from existing studies, without direct involvement of human participants. The primary ethical concern is the potential breach of patient privacy, given that meta-analysis relies on published data and does not involve interventions. To mitigate this risk, we will ensure that all included studies are sourced from legally accessible and publicly available databases, that each study is reviewed for compliance with research ethics, and that the review does not involve individual participant data meta-analysis, thereby ensuring that all data used are anonymized and aggregated. Results will be disseminated through publication in a peer-reviewed journal, presentation at both national and international conferences and forums, and interactive dialogue with indigenous community organizations in the area.

### Study eligibility criteria

#### Inclusion criteria

We will use the Population, Intervention, Comparison, Outcome, and Study type (PICOS) approach to identify eligible studies, with no language restrictions applied. The proposed inclusion criteria for this systematic review are as follows: (1) **Population**: Participants must be medical students who are experiencing workplace bullying, with degree limits including higher vocational education, junior college, undergraduates, postgraduates (master’s or doctoral candidates), and students undergoing resident training; (2) **Intervention**: Workplace bullying must be assessed using the Negative Acts Questionnaire (NAQ) or its revised versions; (3) **Comparison**: Comparisons are not applicable in this study; (4) **Outcome**: Studies must provide prevalence data, including a specific number of participants who are experiencing workplace bullying; (5) **Study Design**: Only cross-sectional studies published before September 1, 2024, will be included.

#### Exclusion criteria

We will exclude studies that meet any of the following criteria: (1) Studies involving secondary vocational school students, nursing students, residents, or junior doctors in a hospital; (2) Studies that do not meet the definition of workplace bullying; (3) Studies that do not specify the measurement tools used; (4) Studies with incorrect data or without separate data for the eligible population. Research providing only prevalence rates or positive number would be excluded; (5) Other study types include intervention studies, reviews, conference papers, qualitative interview studies, longitudinal studies, theses, and dissertations; (6) For studies that contain identical data, only the latest or the most comprehensive one will be included. All others will be excluded; (7) Duplicate publications; (8) Full-text articles are not available after exhaustive searches to locate the texts.

#### Information resources and search strategy

We will comprehensively search general and Chinese databases to identify the relevant literature that meets our criteria from inception to September 1, 2024. The general databases will include PubMed, Embase, and the Web of Science. The Chinese databases will include China National Knowledge Infrastructure (CNKI) and CBM (Sinomed). Our search will utilize medical subject heading (Mesh) terms combined with text words and synonyms, such as ‘medical student’, ‘medical trainee’, ’workplace bullying’, ‘workplace violence’, ‘harassment’, ‘mobbing’, and ‘offending’. The search strategy will be tailored to each database. Additionally, we will perform manual searches and reference checks to expand the search coverage. Details of the search strategy for PubMed database are provided in **S2 File**.

### Outcomes and measurements

#### Primary and secondary outcomes

The primary outcome of the review is the overall prevalence of workplace bullying among medical students, as measured by standard assessment tools. The secondary outcomes are the source of workplace bullying, including superiors, colleagues, and potential influencing factors such as geographic region, gender, specialty, and degree.

#### The definition of a validated measurement tool for our study

In our study, validated measurement tools, namely scales, will be used to assess workplace bullying. Negative Acts Questionnaire (NAQ) and its revision versions will be adapted in our study. NAQ, proposed by Einarsen, is the most widely used instrument worldwide for assessing exposure to workplace bullying [25, 26]. It serves as a reliable measure for evaluation, with diverse translated or revision versions that have undergone rigorous validation, exhibiting strong reliability and validity [27–30]. The NAQ was the initial edition, containing 23 items, and a newer revision followed this in 2009: the Negative Acts Questionnaire-Revised (NAQ-R), evaluating bullying from three dimensions of negative behaviors and a single question [31]. Based on this scale, the prevalence will be measured by self-reported frequency using a 5-point Likert-type scale, ranging from 1 (never) to 5 (daily). Besides the self-reported scales, questionnaires based on NAQ was the most widely used tool when measuring workplace bullying among healthcare workers [32, 33]. We will review the measurement tools used in the literature, including any modified or translated versions, to ensure they have been validated. Relevant reliability and validity data for these tools, such as for the Chinese version of the NAQ-R, will be provided in the meta-analysis. If multiple validated scales are used, we will conduct separate meta-analyses for each distinct scale, if the data is sufficient. Alternatively, we will perform a single integrated meta-analysis that encompasses all scales, considering the scale type as a factor in both subgroup and sensitivity analyses.

#### Study selection and data extraction

Two authors (LHC and MMC) will independently screen the titles and abstracts to assess the eligibility of all studies identified using the abovementioned strategies. Articles for which eligibility is uncertain will be subject to a full-text review to gain more information. These studies will be judged according to the inclusion and exclusion criteria. Assessments will be cross-checked, and discrepancies will be resolved through discussion between the two investigators. If disagreements persist, a third reviewer (CZL) will be consulted to reassess the data and facilitate additional discussions until consensus is achieved. The following data will be extracted using a customized form: study characteristics (e.g., title, first author, year of publication, geographic region, study design, sample size, type of bullying, working conditions); participant characteristics (e.g., gender, age, degree, specialty); the prevalence of workplace bullying; risk factors; and sources of bullying.

#### Risk of bias assessment and study quality

For a meta-analysis of prevalence research, the Joanna Briggs Institute (JBI) critical appraisal checklist for studies reporting prevalence data will be used to evaluate the quality of the studies [34]. This tool assesses studies according to nine items, which cover aspects such as sampling methods, research objects, data collection, and analysis methods (**S3 File)**. Each criterion is assessed as yes, no, unclear, or not applicable. The evaluation strategy is based on JBI working group guidelines [34, 35]. If the answer is yes, then the question is scored as one. Other answers are scored as zero. Total quality scores ≤ 4, 5–7, and ≥ 8 are regarded as low, moderate, and high quality, respectively [36]. Two investigators (LHC and MMC) will independently assess study quality. Their evaluations will be cross-checked, and any discrepancies will be discussed and resolved.

If a consensus cannot be reached, a third reviewer (CZL) will be engaged to re-evaluate the study quality and assist in resolving any persistent disagreements until a final consensus is reached. If more than 10 studies are available, publication bias will be initially analyzed by visually inspecting funnel plots, with roughly symmetrical funnel plots indicating the absence of bias and asymmetrical funnel plots indicating the presence of bias [37]. Following this visual inspection, statistical tests including Begg’s test [38] and Egger’s [39] test will be performed, with a P-value of less than 0.05 indicating potential publication bias. If publication bias is detected, the ’ trim-and-fill method’ will be adopted to adjust for it [40, 41].

### Data analyses

The aggregate prevalence will be pooled in STATA 12.0 software (STATA Corporation, College Station, TX). To assess heterogeneity among the included studies, we will employ the inconsistency index (*I^2^*) and the chi-square test [42, 43]. Both *I^2^* statistic and chi-square test *P* values were used to quantify heterogeneity, with *I^2^* > 50% and P < 0.05 considered as significant cutoffs for heterogeneity. Based on the *I^2^* value, the random-effects model (*I^2^* ≥ 50%, significant heterogeneity) or the fixed-effect model (*I^2^* < 50%, low heterogeneity) will be chosen [42].

To explore the source of heterogeneity, we will perform subgroup analysis, sensitivity analysis, and meta-regression analysis. Subgroup analysis will be conducted based on (1) participant characteristics, including age, gender, race, and academic degree, to assess potential influencing factors, and (2) the type of measurement scale used, to evaluate how different validated scales might affect the results. For the investigation of heterogeneous studies, we plan to conduct sensitive analyses to evaluate the influence of individual studies on the robustness of pooled results as follows: (1) leave-1-out sensitivity analysis; (2) exclusion of low-quality studies according to JBI standards (total quality scores ≤ 4) [34, 44]. The results of the meta-analysis will be considered reliable if the effect size of all studies falls within the 95% confidence interval of the meta-analysis’s effect size, and if there are no discernible differences between the effect size and the total effect size after excluding any study. Given the high likelihood of heterogeneity in prevalence studies, if applicable, we will further perform a meta-regression analysis to investigate possible sources of heterogeneity [45].

## Discussion

This protocol is for our meta-analysis on the prevalence of workplace bullying in global medical students, a topic that has not received sufficient attention to date. Published studies conducted by certain hospitals indicate that the prevalence of workplace bullying among medical students varies internationally. Thus, through meta-analysis, we aim to determine the overall severity of workplace bullying and identify potential influencing factors. Our findings could provide valuable insights for educators, healthcare managers, and policymakers. By understanding the scope and factors contributing to workplace bullying, they can develop targeted precautions and interventions to address this issue. Ultimately, our goal is to enhance the health quality and occupational environment for medical students, thereby supporting their professional development and well-being.

## Supporting information

S1 File PRISMA-P 2015 checklist. (DOCX)

S2 File PubMed search strategy. (DOCX)

S3 File JBI critical appraisal checklist for studies reporting prevalence data. (DOCX)
