# Supplementary material for: Prevalence of workplace bullying among medical students: A meta-analysis and systematic review protocol
Source: PLoS One. 2025 Feb 13;20(2):e0310076. doi: 10.1371/journal.pone.0310076 (PMC11824983; doi:10.1371/journal.pone.0310076)
Supplement: S2 File — (DOCX) [file pone.0310076.s002.docx]

**PubMed Search Strategy**

**#1** (((((((medical students [MeSH Terms]) OR (medical trainees [Title/Abstract])) OR (medical learners [Title/Abstract])) OR (medical interns [Title/Abstract])) OR (medical residents [Title/Abstract])) OR (medical fellows [Title/Abstract])) OR (medical pupils [Title/Abstract])) OR (medical apprentices [Title/Abstract])

**#2** ((((((((workplace bullying [MeSH Terms]) OR (harassment [Title/Abstract])) OR (aggression [Title/Abstract])) OR (mistreatment [Title/Abstract])) OR (victimization [Title/Abstract])) OR (abuse [Title/Abstract])) OR (intimidation [Title/Abstract])) OR (incivility [Title/Abstract])) OR (violence [Title/Abstract])

**#3** ('Negative Acts Questionnaire'[All fields]) OR (NAQ [All fields])

**#4** #1 AND #2 AND #3
